# Validation of a Portable Game Controller to Assess Peak Expiratory Flow Against Conventional Spirometry in Children: Cross-sectional Study

**DOI:** 10.2196/25052

**Published:** 2021-01-29

**Authors:** Khadidja Chelabi, Fabio Balli, Myriam Bransi, Yannick Gervais, Clement Marthe, Sze Man Tse

**Affiliations:** 1 Faculty of Medicine McGill University Montreal, QC Canada; 2 Breathing Games Association Geneva Switzerland; 3 Milieux Institute Concordia University Montreal, QC Canada; 4 Faculty of Medicine Laval University Quebec, QC Canada; 5 Department of Pediatrics Centre mère-enfant Soleil du CHU de Québec Quebec, QC Canada; 6 Division of Respiratory Medicine Department of Pediatrics Sainte-Justine University Hospital Center Montreal, QC Canada; 7 Faculty of Medicine University of Montreal Montreal, QC Canada

**Keywords:** asthma, pediatrics, serious game, peak expiratory flow, pulmonary function test, adherence, self-management

## Abstract

**Background:**

International asthma guidelines recommend the monitoring of peak expiratory flow (PEF) as part of asthma self-management in children and adolescents who poorly perceive airflow obstruction, those with a history of severe exacerbations, or those who have difficulty controlling asthma. Measured with a peak flow meter, PEF represents a person’s maximum speed of expiration and helps individuals to follow their disease evolution and, ultimately, to prevent asthma exacerbations. However, patient adherence to regular peak flow meter use is poor, particularly in pediatric populations. To address this, we developed an interactive tablet-based game with a portable game controller that can transduce a signal from the user’s breath to generate a PEF value.

**Objective:**

The purpose of this study was to evaluate the concordance between PEF values obtained with the game controller and various measures derived from conventional pulmonary function tests (ie, spirometry) and to synthesize the participants’ feedback.

**Methods:**

In this cross-sectional multicenter study, 158 children (aged 8-15 years old) with a diagnosis or suspicion of asthma performed spirometry and played the game in one of two hospital university centers. We evaluated the correlation between PEF measured by both the game controller and spirometry, forced expiratory volume at 1 second (FEV_1_), and forced expiratory flow at 25%-75% of pulmonary volume (FEF_25-75_), using Spearman correlation. A Bland-Altman plot was generated for comparison of PEF measured by the game controller against PEF measured by spirometry. A post-game user feedback questionnaire was administered and analyzed.

**Results:**

The participants had a mean age of 10.9 (SD 2.5) years, 44% (71/158) were female, and 88% (139/158) were White. On average, the pulmonary function of the participants was normal, including FEV_1_, PEF, and FEV_1_/forced vital capacity (FVC). The PEF measured by the game controller was reproducible in 96.2% (152/158) of participants according to standardized criteria. The PEF measured by the game controller presented a good correlation with PEF measured by spirometry (*r*=0.83, *P*<.001), with FEV_1_ (r=0.74, *P*<.001), and with FEF_25-75_ (*r*=0.65, *P*<.001). The PEF measured by the game controller presented an expected mean bias of –36.4 L/min as compared to PEF measured by spirometry. The participants’ feedback was strongly positive, with 78.3% (123/157) reporting they would use the game if they had it at home.

**Conclusions:**

The game controller we developed is an interactive tool appreciated by children with asthma, and the PEF values measured by the game controller are reproducible, with a good correlation to values measured by conventional spirometry. Future studies are necessary to evaluate the clinical impact this novel tool might have on asthma management and its potential use in an out-of-hospital setting.

## Introduction

Asthma is a chronic pulmonary disorder that affects more than 339 million people globally [1]. It is the world’s most prevalent chronic disease in children, affecting 7.5% of children in the US [2], and can have a significant impact on one’s life. Indeed, asthma attacks result in 146,000 annual visits to emergency departments and are the primary cause for school absence [3]. Characterized by reversible airway narrowing and chronic inflammation, asthma is a heterogeneous disease that can present with diverse respiratory symptoms, which can be periodically exacerbated by personal triggers [4,5]. While recognition of asthma symptoms is key to managing and preventing asthma exacerbations, self-perception and self-reporting of symptoms by the affected individuals is often difficult and unreliable [6]. This is particularly problematic in children. As such, international guidelines [7-9] recommend self-monitoring of PEF in the asthmatic population starting at 5 years of age, more specifically in children and adolescents who poorly perceive airflow obstruction, those with a history of severe exacerbations, or those who have difficulty controlling asthma. While symptoms-based asthma action plans have been shown to be superior to peak flow–based plans, peak flow may be used as a complementary measure to monitor symptoms in self-management of asthma. In fact, studies [10-12] have effectively shown that daily measurement of PEF in children as a self-monitoring tool can increase adherence to medications and decrease asthma exacerbations in children with poor symptoms perception. Despite this evidence, there is a low adherence to PEF monitoring using current peak flow meters in the pediatric population, with a reported 15% adherence to the Mini Wright Peak Flow Meter in children only 3 weeks after initial use [13]. Some of the identified barriers to PEF monitoring are the self-perception that one’s asthma is well-controlled, the perceived burden of taking frequent measurements with seemingly low direct benefit to one’s general health, and the financial barriers to purchasing a peak flow meter, with the cost ranging from 20-50 Canadian dollars per device (US $15.73-$39.33) [14]. This issue of low adherence to PEF monitoring in pediatric asthma needs to be addressed in order to engage and empower children early in healthy management of their chronic illness, improve their ability to perceive symptoms, and potentially decrease exacerbations.

Serious games, defined as games with a primary purpose other than pure entertainment [15], can be a means to increase adherence to treatments or therapies in chronic diseases and have been associated with positive health outcomes [16-18]. Serious games may be used to actively engage patients in their health management in a playful setting and are of particular use to children, given the gaming approach. Although several serious games have addressed the goal of knowledge acquisition in asthma [19], none has yet suggested a serious game approach to self-monitoring of asthma symptoms, including the measurement of PEF. Importantly, airflow measurements taken through serious games need to be validated against standard measures before the games can be implemented clinically.

In this study, we evaluated the validity of an airflow-based game controller paired with the *TikiFlow* game for portable electronic devices to monitor PEF in children with asthma using conventional spirometry as the comparison standard. We evaluated the concordance between PEF measurements obtained with the game controller and several spirometry measurements, and the reproducibility of PEF measured by the game controller without coaching. Furthermore, we assessed the participants’ feedback both quantitatively and qualitatively to guide the continuous development of the game.

## Methods

### Game Controller and Game Design

**Figure 1:**
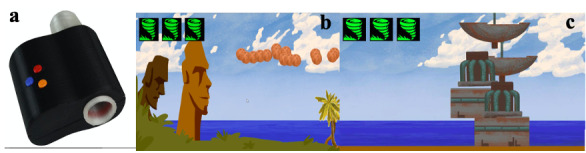
Representation of the game controller and the TikiFlow game. Children forcefully exhale in the mouthpiece of the game controller (a) to propel plant seeds, water them, and repopulate Tiki island with up to 5 attempts in the Tikiflow tablet-based game (b,c). The peak expiratory flow results appear at the end of the game. Images are copyrighted to breathinggames.net.

We designed a 3D-printed game controller to transduce the user’s breath into a digital signal and to meet the calibration standards of the Omron PF9940 peak flow meter. The game controller uses Bluetooth to connect to a portable electronic device and allows control of the *TikiFlow* game by the means of airflow through the game controller (Figure 1). The *TikiFlow* game challenges the participant to exert a forceful expiration in order to propel plant seeds, trigger a rainfall, and repopulate an island destroyed by pollution. PEF results measured by the game controller appear at the end of the game. The game can be previewed online [20].

### Design of the Study

We conducted a cross-sectional study on children who presented to the pulmonary function test laboratory of two pediatric university health centers in Quebec, Canada (Centre Hospitalier Universitaire Sainte-Justine and Centre Hospitalier Universitaire de Québec – Université Laval) over a 3-week period in November 2019. The respective institutional research ethics board approved this study (Centre Hospitalier Universitaire Sainte-Justine #MP-21-2020-2310, Centre Hospitalier Universitaire de Québec-Université Laval #MEO-21-2020-4711 and Concordia University #30011659).

### Study Population

We included children aged 8-15 years old with a diagnosis of asthma or a suspicion of asthma presenting to pulmonary function test laboratory for a spirometry, with or without bronchodilator reversibility testing, at their physician’s request. We excluded children with cystic fibrosis, bronchopulmonary dysplasia, and other conditions where the indication for pulmonary function test was unrelated to asthma (eg, follow-up of oncology patients or esophageal atresia). We also excluded children who were unable to cooperate for either conventional spirometry or assessment via the game controller according to American Thoracic Society guidelines. We identified eligible participants through the pulmonary function test laboratory database of both pediatric university health centers.

### Measurements

We asked eligible and consenting participants to play *TikiFlow* with the game controller without coaching within 10 minutes of their conventional spirometry. Specifically, participants were given a brief overview of the game and its purpose and were asked to play on their own without instructions to exert more forceful exhalations or to increase the effort of breathing. Following the game, they also filled out a self-reported general health questionnaire and a satisfaction questionnaire.

Conventional spirometry measurements were obtained from a Jaeger MasterScope spirometer (Cardinal Health, Dublin, OH), using the Global Lung Initiative 2012 reference values [21] and interpreted according to American Thoracic Society guidelines [22]. Specifically, we retained the forced expiratory volume at 1 second (FEV_1_), the forced expiratory flow at 25%-75% of pulmonary volume (FEF_25-75_), and the forced vital capacity values from the participants’ best curve, and the highest PEF value (PEF measured by spirometry). For PEF measured by spirometry, we evaluated the reproducibility of the spirometry in accordance with the American Thoracic Society guidelines, defined as the 2 highest values falling within 40L/min of each other [22]. For PEF measured by the game controller, we established a minimum of 3 reproducible forced exhalations as necessary, of which 2 must meet the American Thoracic Society reproducibility criteria, in order to end the game. The game was stopped if reproducibility could not be achieved after 5 exhalations. If the child was prescribed a reversibility testing with bronchodilators, which consists of repeating the spirometry 15 minutes following the administration of the bronchodilator salbutamol, we repeated the PEF game controller measurements following the post-bronchodilator spirometry. We collected the participant’s appreciation of the game through a 5-question Likert-scale questionnaire and narrative feedback.

### Statistical Analysis

We portrayed a descriptive analysis of the participants’ baseline health and demographics based on the results of the self-reported questionnaire. A Bland-Altman plot was used to compare the game controller method to that of conventional spirometry and to evaluate the concordance of both methods to measure PEF. We calculated the spearman correlation between PEF measured by the game controller and by conventional spirometry measures. Statistical significance was set at *P*<.05. Analyses were performed with R software, version 3.5.0 [23]. Participant’s appreciation of the game was evaluated quantitatively based on the satisfaction questionnaire administered and results were plotted in a bar graph.

## Results

### Population

We identified 337 children aged 8-15 years old presenting to both centers for a spirometry, with or without bronchodilator reversibility testing, 187 of whom were eligible based on inclusion criteria. We included 158 participants, as 9 were unable to perform spirometry and 20 refused to take part in the study, mostly due to time constraints (see Multimedia Appendix 1).

Participants had a mean age of 10.9 (SD 2.5) years (Table 1), and most were male (88/158, 55.7%), a proportion that reflects the sex distribution in pediatric asthma in the general population [24]. The majority were White (139/158, 88.0%). Up to 68% (108/158) of participants presented an atopic profile, defined as also having eczema or food or environmental allergies. The characteristics of participants were similar between both centers (see Multimedia Appendix 2).

Almost all participants (156/158, 98.7%) performed a reproducible spirometry. On average, the baseline lung function of participants was normal, with a mean percent predicted value of 103.7% (SD 11.7%) for forced vital capacity, 99.6% (SD 13.2%) for FEV_1_, and 86.2% (SD 25.5%) for FEF_25-75_. However, varying degrees of airway obstruction are represented in our participants as demonstrated by the range of percent predicted values and z scores (Table 2; see Multimedia Appendix 3).

**Table 1:** Selected baseline characteristics of participants (n=158).

Characteristic		Value
Age, mean years (SD)		10.9 (2.5)
Male, n (%)		88 (55.7)
BMI percentile, median (IQR)		49 (33.3-90.8)
**Ethnicity, n (%)**		
	White	139 (88.0)
	Black	11 (6.8)
	Other	8 (5.1)
Diagnosis of asthma, n (%)		131 (82.9)
Age at diagnosis, mean years (SD)		4.2 (3.2)
**Daily controller therapy, n (%)**		133 (84.2)
	ICS^a^ only	58 (36.7)
	ICS-LABA^b^	33 (20.9)
	ICS-LTRA^c^	11 (7.0)
	ICS-LABA-LTRA^d^	27 (17.1)
	LTRA^e^ only	4 (2.5)
Eczema, n (%)		45 (28.5)
Food allergy, n (%)		38 (24.1)

^a^ICS: inhaled corticosteroids.

^b^ICS-LABA: inhaled corticosteroids and long acting beta-agonist.

^c^ICS-LTRA: inhaled corticosteroids and leukotriene receptor antagonist.

^d^ICS-LABA-LTRA: inhaled corticosteroids, long acting beta-agonist and leukotriene receptor antagonist.

^e^LTRA: leukotriene receptor antagonist.

**Table 2:** Baseline lung function assessed by conventional spirometry (n=158).

Function		Value
**FVC^a^**		
	% predicted, mean (SD)	103.7 (11.7)
	% predicted, range	66.5 to 142.9
	z score, mean (SD)	0.3 (1.0)
	z score, range	–3.0 to 3.5
**FEV_1_^b^**		
	% predicted, mean (SD)	99.6 (13.2)
	% predicted, range	59.3 to 130.2
	z score, mean (SD)	0.0 (1.1)
	z score, range	–3.4 to 2.3
**FEF_25-75_^c^**		
	% predicted, mean (SD)	86.2 (25.5)
	% predicted, range	20.8 to 184.8
	z score, mean (SD)	–0.7 (1.2)
	z score, range	–4.1 to 3.6
FEV_1_/FVC^d^, mean (SD)		83.6 (6.8)
PEF_spiro_^e^ (L/min), median percentile (IQR)		266.7 (230.1-336.9)

^a^FVC: forced vital capacity.

^b^FEV_1_: forced expiratory volume in 1 second.

^c^FEF_25-75_: forced expiratory flow at 25%-75% of the pulmonary volume.

^d^FEV_1_/FVC: forced expiratory volume in 1 second/forced vital capacity.

^e^PEF_spiro_: PEF measured by spirometry.

### Comparison of the Game Controller and Conventional Spirometry

**Figure 2:**
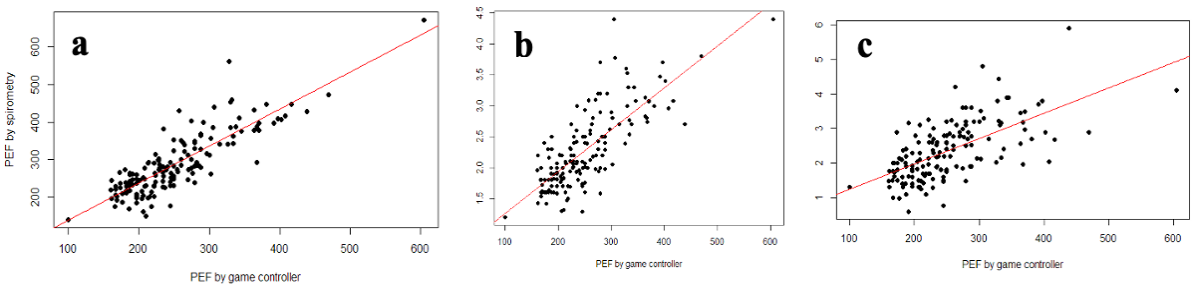
Correlation between PEFGC and selected conventional spirometry measurements. PEFGC shows good correlation with PEFspiro (a; *r*=0.83, *P*<.001), FEV_1_ (b; *r*=0.74, *P*<.001) and FEF_25-75_ (c; *_25-75_*=0.65, *P*<.001). PEFGC: peak expiratory flow measured by the game controller. PEFspiro: peak expiratory flow measured by conventional spirometry. FEV_1_: forced expiratory volume in 1 second. FEF_25-75_: forced expiratory flow at 25%-75% pulmonary volume.

**Figure 3:**
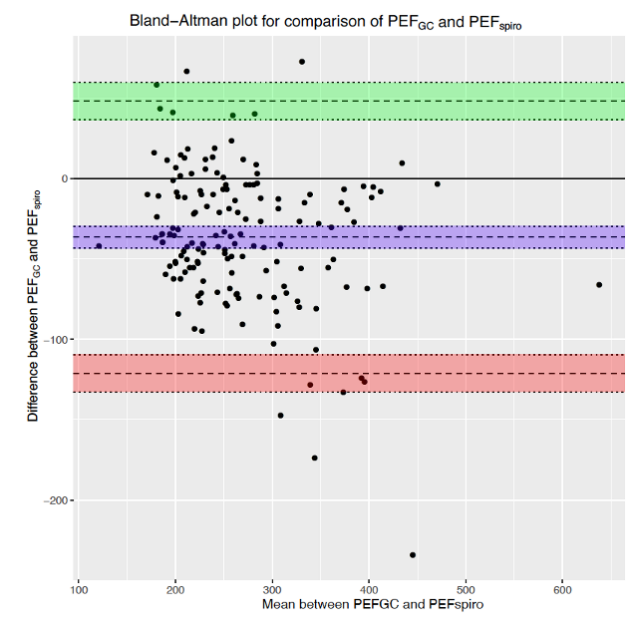
Bland-Altman plot comparing the game controller and conventional spirometry for PEF measurements. The game controller presents a mean bias of –36.4 L/min (blue; 95% CI –43.2 to –29.6) with a 95% upper limit of agreement of 48.3 (green; 95% CI 36.6 to 60.0) and a 95% lower limit of agreement of –121.0 (red; 95% CI –132.7 to –109.4) when compared to conventional spirometry. PEF: peak expiratory flow. PEF*GC*: peak expiratory flow measured by the game controller. PEF*spiro*: peak expiratory flow measured by conventional spirometry.

Despite the lack of coaching, 96.2% (152/158) of PEF values measured by the game controller were reproducible. Spearman correlations indicated that PEF measured by the game controller presented a good correlation with PEF measured by spirometry (*r*=0.83, *P*<.001), with FEV_1_ (*r*=0.74, *P*<.001), and with FEF_25-75_ (*r*=0.65, *P*<.001) (Figure 2). The Bland-Altman plot showed that the PEF measured by the game controller presented a mean bias of –36.4 L/min (95% CI –43.2 to 29.6) compared to PEF measured by spirometry. The 95% limits of agreement, defined as the values within which 95% of differences between the two measurement methods lay, were –121.0 and 48.3 L/min (Figure 3). We identified 5 outliers, whose exclusion in a sensitivity analysis did not result in significant change in the concordance or correlation measures.

Of the 84 participants who underwent a spirometry pre- and post-bronchodilator administration, 7 were considered to have reversible airway obstruction as defined by a change in FEV_1_ of ≥12% post-bronchodilator [25]. Using the game controller, 4 of those 7 participants were considered to have reversible obstruction as defined by a change in PEF measured by the game controller of ≥15%[26]. Based on these limited numbers, we could not reliably assess the responsiveness of PEF measured by the game controller to bronchodilator as compared to conventional spirometry.

### Appreciation Feedback of the Game Controller

**Figure 4:**
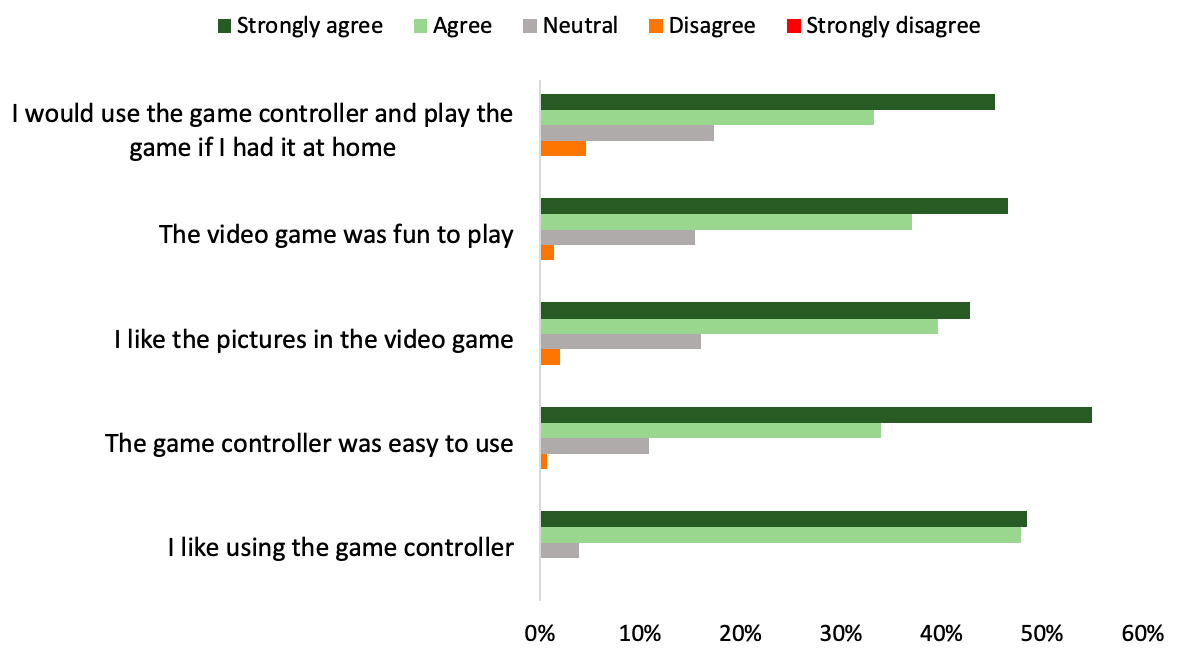
Game appreciation as reported by the participants.

Overall, users highly appreciated the game (Figure 4). No participant reported “Strongly disagree” to any of the 5 statements, and 78.3% (123/157) of participants reported they would use the game controller and play the game if they had it at home. 107 participants provided positive narrative feedback from which we noted that the main positive aspects of the game were the game concept (n=31), its educational aspect coupled with its entertaining aspect (n=14), and the ease of use of the game controller (n=25). Amongst the 28 youngest participants, aged 8-11 years old, who provided feedback on areas of improvement, the most recurrent comment was the desire for a longer duration to the game.

Of the 21 participants that gave negative narrative feedback, we noted the older users (aged 12-15 years) sometimes felt that the game content was intended for a younger population (n=6).

## Discussion

### Principal Findings

This study shows that the PEF measurements obtained through our game controller present a good correlation with conventional spirometry measurements and have a high reproducibility. Additionally, the participants reported overall strongly positive feedback on the game controller and the game, with 78.3% (123/157) reporting that they would play the game if they had it at home. These findings suggest that our game and controller could provide an objective measure of airway obstruction and that its use in an out-of-hospital setting could be explored.

PEF self-monitoring has been shown to reduce asthma exacerbations through the development of asthma awareness and control [10–12]. However, despite the international asthma guidelines recommendations for its adoption in poor symptoms perceivers and those with difficult to control asthma, few physicians educate and recommend PEF monitoring to their patients [27,28]. Two of the reasons for clinicians’ low advocacy for peak flow meter use are the wide intraindividual variability in PEF values [29] and the low adherence to their use, particularly in pediatric patients with asthma.

Specifically, peak flow meters do not seem to consistently report PEF values that correlate with conventional spirometry, and a different measurement bias is associated with each peak flow meter [30,31]. Our game controller underestimated PEF measured by spirometry by 36.4 L/min, which is comparable to other peak flow meters [31-33]. This bias is expected as differing breathing techniques are used for PEF (child breathes out forcefully without coaching) and spirometry (child breathes out forcefully and completely with coaching) [31]. Reassuringly, our findings suggest a good correlation between PEF measured by the game controller and various spirometry measures across different degrees of baseline airway obstruction, suggesting that PEF measured by the game controller could be used as a surrogate to monitor trends in lung function. Furthermore, game controller measures were reproducible in 96.2% (152/158) of participants despite the lack of coaching, likely facilitated by the intuitiveness of the game narrative and mechanics. This finding suggests that PEF self-monitoring with our game controller would be feasible in a nonclinical environment without the supervision of a health care professional. Although we are not advocating for peak flow to replace symptoms-based asthma action plans, we believe that the addition of this objective measure in select patients can help with asthma control. This feature could have potentially significant benefits, namely by reducing barriers to health care access and reducing costs and transportations, all of which need to be further studied.

One suggested approach to increase adherence to medical treatments and to engage and empower the population in management of diseases is the use of serious games. Serious games have found their utility in different disciplines of medicine, whether to distract patients from a painful procedure, to engage adolescents in psychotherapies, or to educate patients on complex medical topics. Importantly, studies report a high adherence of serious games whether at home or in clinical settings [17-19]. Only 5 of 187 (2.7%) eligible children refused to participate due to a lack of interest, suggesting that *TikiFlow* appealed to the vast majority of children (see Multimedia Appendix 1). Additionally, more than 75% of the participants reported that they enjoyed the game (150/156), that it was fun to play (131/157), easy to use (139/157), and that they would use it at home (123/157). These findings were independent of age or sex.

Another approach to increase adherence specifically to PEF monitoring in asthma is the automatic generation of diaries as opposed to the traditional handwritten personal diaries of PEF measures. This approach decreases the effort required by the user and reduces the risk of false data generation. It also offers the possibility to depict measures graphically over time to identify trends. A recent study in the United Kingdom [34] suggests smartphone self-generated PEF diaries alone increase compliance with up to 32% of adults taking at least one PEF measurement daily for 3 months after the initiation of the study and 28% after 6 months. Our game takes advantage of this approach and integrates an automatically generated diary of PEF measures.

Serious games may be particularly beneficial among children with chronic conditions, where they were found to improve knowledge level and self-management [18]. The role of serious games in asthma self-management has been little explored. Asthma-related serious games, to date, have focused heavily on asthma education, and while they seem to improve knowledge transfer, they have been found to have little to no effect on behavior modification [19]. The novelty in our game, *TikiFlow*, is the use of a game controller with which the child uses their breath to control the game, bringing the focus to the play, rather than education alone. The acquisition of good asthma self-care habits early in childhood and adolescence may translate into long-lasting benefits in adulthood [35].

One notable strength of our game is its accessibility. Asthma is a global disease and the development of innovative tools to help patients and their families in their asthma management must be accessible to individuals in low- and middle-income countries. Smartphones and tablets represent ideal mediums to this end, as 66% of the world’s population has access to such a device [36]. Our game and game controller are open source and our controller is 3D-printed, which makes it easy to reproduce at low cost and with minimal equipment, reducing access barriers. Furthermore, its small size and its portable format makes it easy to carry and incorporate in daily activities and, importantly, easy to disinfect.

There are several limitations to this study. First, our study does not include children aged 5-8 years old despite the international asthma guidelines recommending PEF monitoring in children aged ≥5 years old [8,37]. Our decision was to limit the confounders of poor effort and lack of cooperation in young children in the assessment of the game controller’s validity. Further studies are necessary to evaluate the feasibility of PEF game controller measurements in this age group. Second, our study was conducted in tertiary care centers with a relatively healthy population with average normal functions at baseline. Thus, our results cannot be generalized to individuals with lower lung function or to those who are in acute exacerbations. Third, all game controller measurements were done systematically after conventional spirometry. Therefore, it is possible that fatigue contributed to skewing the game controller’s measurements to lower values in our study. Participants were given instructions for the conventional spirometry prior to the game, which may have motivated a better effort on the game controller than could be expected with home measurements. However, we explicitly did not give instructions for the use of the game in order to reproduce the home environment as much as possible. Because we only had a few participants with documented reversibility to bronchodilator, we were not able to evaluate the responsiveness of PEF measured by the game controller to bronchodilator. Future studies are needed to explore the characteristics of PEF measured by the game controller and whether it can help predict asthma exacerbations.

### Conclusions

Our study evaluated the use of a novel airflow-based game controller coupled with a smart device serious game in children with asthma. Our game controller produced reproducible PEF measures that correlated well with several conventional spirometry measures and was highly appreciated by participants. Thus, our game controller, coupled with a serious game, may have the potential to increase adherence to PEF self-monitoring in children. Further studies are necessary to evaluate users’ adherence over time, the validity of the game controller in different situations, and its impact on clinical outcomes, specifically its role in the prevention of asthma exacerbations.

## Appendix

Multimedia Appendix 1 Flowchart of patient enrolment in the study.

Multimedia Appendix 2 Baseline characteristics of participants.

Multimedia Appendix 3 Baseline lung function assessed by conventional spirometry.

## Abbreviations

FEF_25-75_: forced expiratory flow at 25%-75% of pulmonary volume
FEV_1_: forced expiratory volume at 1 second
FVC: forced vital capacity
PEF: peak expiratory flow

## References

[1] Asthma World Health Organization Internet. WHO.

[2] Most Recent National Asthma Data | CDC Internet. Center for Disease Control and Prevention.

[3] National Lung Health Framework 2008.

[4] Papi A, Brightling C, Pedersen SE, Reddel HK (2018). Asthma. The Lancet.

[5] van den Wijngaart LS, Roukema J, Merkus PJ (2015). Respiratory disease and respiratory physiology: Putting lung function into perspective: Paediatric asthma. Respirology.

[6] Still L, Dolen WK (2016). The Perception of Asthma Severity in Children. Curr Allergy Asthma Rep.

[7] Global Lung Initiative for Asthma 2020.

[8] National Asthma Education and Prevention Program (2007). Expert Panel Report 3 (EPR-3): Guidelines for the Diagnosis and Management of Asthma–Summary Report 2007. Journal of Allergy and Clinical Immunology.

[9] Zemek Roger L, Bhogal Sanjit Kaur, Ducharme Francine M (2008). Systematic review of randomized controlled trials examining written action plans in children: what is the plan?. Arch Pediatr Adolesc Med.

[10] Feldman JM, Kutner H, Matte L, Lupkin M, Steinberg D, Sidora-Arcoleo K, Serebrisky D, Warman K (2012). Prediction of peak flow values followed by feedback improves perception of lung function and adherence to inhaled corticosteroids in children with asthma. Thorax.

[11] Burkhart PV, Rayens MK, Oakley MG, Abshire DA, Zhang M (2007). Testing an Intervention to Promote Children's Adherence to Asthma Self-Management. J Nursing Scholarship.

[12] Paton JY (2012). Perception of lung function, adherence to inhaled corticosteroids, and the role of peak expiratory flow feedback in paediatric asthma. Thorax.

[13] Redline S, Wright EC, Kattan M, Kercsmar C, Weiss K (1996). Short-term compliance with peak flow monitoring: Results from a study of inner city children with asthma. Pediatr. Pulmonol.

[14] Harver A, Humphries CT, Kotses H (2009). Do Asthma Patients Prefer to Monitor Symptoms or Peak Flow?. Journal of Asthma.

[15] Chen S, Michael D (2006). Serious Games: Games That Educate, Train, and Inform. USA: Thomson Course Technology; 271 p.

[16] Lopes S, Magalhães P, Pereira A, Martins J, Magalhães C, Chaleta E, Rosário P (2018). Games Used With Serious Purposes: A Systematic Review of Interventions in Patients With Cerebral Palsy. Front. Psychol.

[17] Eichenberg C, Schott M (2017). Serious Games for Psychotherapy: A Systematic Review. Games for Health Journal.

[18] Charlier N, Zupancic N, Fieuws S, Denhaerynck K, Zaman B, Moons P (2016). Serious games for improving knowledge and self-management in young people with chronic conditions: a systematic review and meta-analysis. J Am Med Inform Assoc Jan;?.

[19] Drummond D, Monnier D, Tesnière A, Hadchouel A (2017). A systematic review of serious games in asthma education. Pediatr Allergy Immunol.

[20] (2019). TikiFlow game preview Internet. Breathing Games.

[21] GLI Spirometry-Normal Values.

[22] Miller MR (2005). Standardisation of spirometry. European Respiratory Journal.

[23] R project.

[24] Shah R, Newcomb D (2018). Sex Bias in Asthma Prevalence and Pathogenesis. Front Immunol Internet Dec.

[25] Pellegrino R (2005). Interpretative strategies for lung function tests. European Respiratory Journal Nov 1;?.

[26] GINA guidelines 2020.

[27] Akinbami L, Salo P, Cloutier M, Wilkerson J, Elward K, Mazurek J (2019). Primary care clinician adherence with asthma guidelines: the National Asthma Survey of Physicians. Journal of Asthma. 2020 May 3;?.

[28] Cabana M, Rand C, Becher O, Rubin H (2001). Reasons for Pediatrician Nonadherence to Asthma Guidelines. Arch Pediatr Adolesc Med Sep 1;?.

[29] Hegewald M, Crapo R, Jensen R (1995). Intraindividual Peak Flow Variability. CHEST Jan 1;?.

[30] Miller M, Dickinson S, Hitchings D (1992). The accuracy of portable peak flow meters. Thorax Nov;?.

[31] Bongers T, O?Driscoll B (2006). Effects of equipment and technique on peak flow measurements. BMC Pulm Med Dec.

[32] Pesola G, O?Donnell P, Pesola G, Chinchilli V, Saari A (2009). Peak expiratory flow in normals: comparison of the mini Wright versus spirometric predicted peak flows. J Asthma Oct;?.

[33] Kp J, Ma M (1990). Measuring peak expiratory flow in general practice: comparison of mini Wright peak flow meterturbine spirometer. BMJ Jun 1;?.

[34] Antalffy T, De SA, Griffiths C (2020). Promising peak flow diary compliance with an electronic peak flow meter and linked smartphone app. npj Primary Care Respiratory Medicine. 00 May 8;?.

[35] Roberts G, Vazquez-Ortiz Marta, Knibb R, Khaleva E, Alviani C, Angier E, Blumchen Katharina, Comberiati Pasquale, Duca Bettina, DunnGalvin Audrey, Garriga-Baraut Teresa, Gore Claudia, Gowland M Hazel, Hox Valérie, Jensen Britt, Mortz Charlotte G, Pfaar Oliver, Pite Helena, Santos Alexandra F, Sanchez-Garcia Silvia, Timmermans Frans (2020). EAACI Guidelines on the effective transition of adolescents and young adults with allergy and asthma. Allergy.

[36] DataReportal Global Digital Insights Digital 2020: July Global Statshot Internet.

[37] GINA guidelines 2016.

[38] Source code of TikiFlow.

[39] Design of the game controller for TikiFlow.

