# Supplementary figures and images for: Validation of a Portable Game Controller to Assess Peak Expiratory Flow Against Conventional Spirometry in Children: Cross-sectional Study
Source: JMIR Serious Games. 2021 Jan 29;9(1):e25052. doi: 10.2196/25052 (PMC7880812; doi:10.2196/25052)

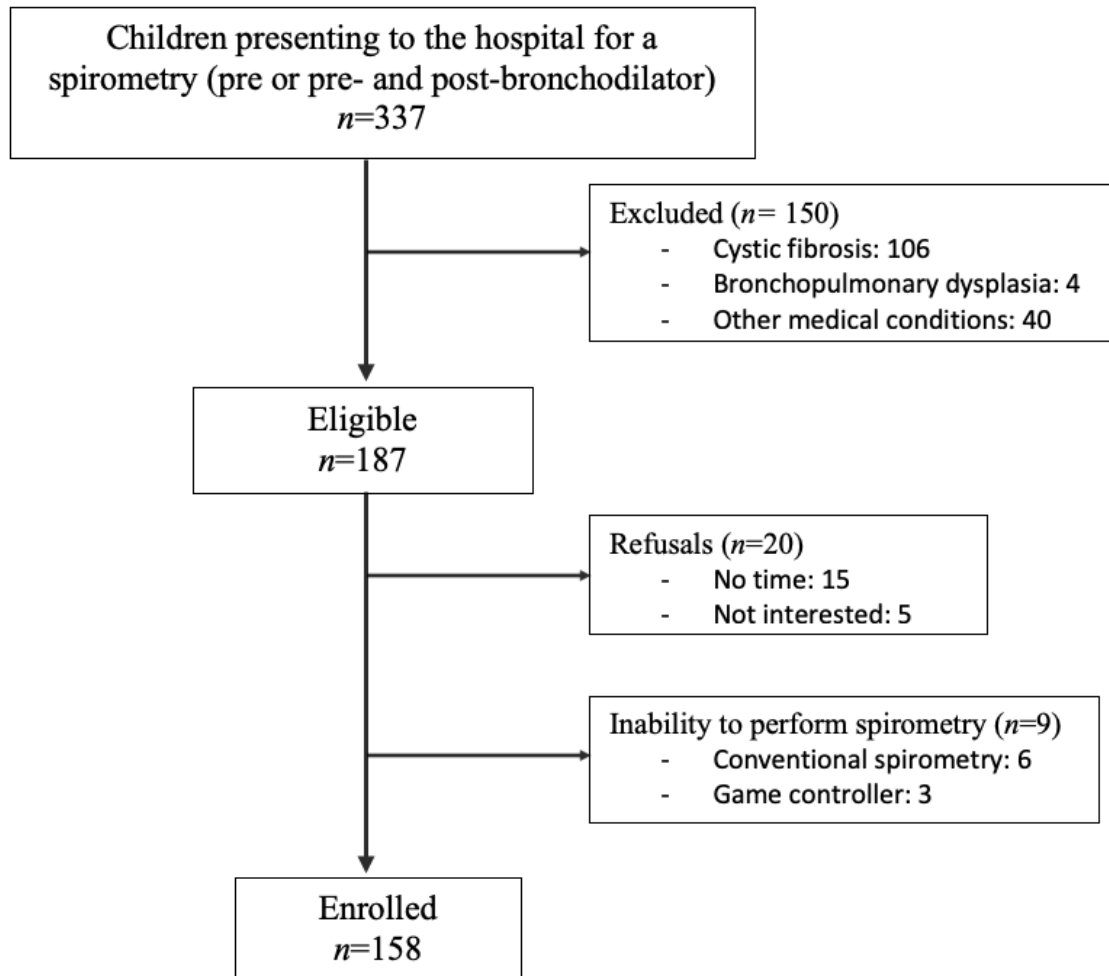

Multimedia appendix 1. **Flowchart of patient enrolment in the study**

Supplement: Multimedia Appendix 1 [file games_v9i1e25052_app1.pdf]
