# Supplementary material for: Validation of a Portable Game Controller to Assess Peak Expiratory Flow Against Conventional Spirometry in Children: Cross-sectional Study
Source: JMIR Serious Games. 2021 Jan 29;9(1):e25052. doi: 10.2196/25052 (PMC7880812; doi:10.2196/25052)
Supplement: Multimedia Appendix 2 [file games_v9i1e25052_app2.pdf]

Multimedia appendix 2-Baseline characteristics of participants			
	**CHUSJ (N=88)**	**CHUQ (N=70)**	**Total (N=158)**
Age, years, mean (SD)	10.7 (2.4)	11.0 (2.5)	10.9 (2.5)
Male, n (%)	50 (56.8)	38 (54.3)	88 (55.7)
BMI, percentile, median (IQR)	73 (38.3, 93.0)	57 (27.5, 80.0)	49 (33.3, 90.8)
Ethnicity, n (%)			
Caucasian	69 (78.4)	70 (100)	139 (88.0)
Black	11 (12.5)	0 (0)	11 (6.8)
Other	8 (9.1)	0 (0)	8 (5.1)
Diagnosis of asthma, n (%)	71 (80.7)	60 (85.7)	131 (82.9)
Age at diagnosis, years, mean (SD)	4.1 (3.5)	4.4 (2.6)	4.2 (3.2)
Daily controller therapy, n (%)			
ICS only	32 (36.4)	26 (37.1)	58 (36.7)
ICS-LABA	15 (17.0)	18 (25.7)	33 (20.9)
ICS-LTRA	7 (8.0)	4 (5.7)	11(7.0)
ICS-LABA-LTRA	13 (14.8)	14 (20.0)	27 (17.1)
LTRA only	2 (2.3)	2 (2.9)	4 (2.5)
Eczema, n (%)	32 (36.4)	13 (18.6)	45 (28.5)
Food allergy, n (%)	24 (27.3)	14 (20.0)	38 (24.1)
Environmental allergy: *n* (%)	61 (69.3)	47 (67.1)	108 (68.4)

CHUSJ: Sainte-Justine hospital university center, CHUQ: Quebec hospital university center, BMI: body mass index, ICS: inhaled corticosteroids, ICS-LABA: inhaled corticosteroids and long acting beta-agonist, ICS-LTRA: inhaled corticosteroids and leukotriene receptor antagonist, ICS-LABA-LTRA: inhaled corticosteroids and long acting beta-agonist and leukotriene receptor antagonist, LTRA: leukotriene receptor antagonist, SD: standard deviation, IQR: interquartile range
