# Supplementary material for: Validation of a Portable Game Controller to Assess Peak Expiratory Flow Against Conventional Spirometry in Children: Cross-sectional Study
Source: JMIR Serious Games. 2021 Jan 29;9(1):e25052. doi: 10.2196/25052 (PMC7880812; doi:10.2196/25052)
Supplement: Multimedia Appendix 3 [file games_v9i1e25052_app3.pdf]

Multimedia appendix 3-Baseline lung function assessed by conventional spirometry			
	**CHUSJ (N=88)**	**CHUQ (N=70)**	**Total (N=158)**
FVC:			
% predicted, mean (SD)	106.1 (12.3)	101.0 (10.3)	103.7 (11.7)
% predicted, range	66.5, 142.9	79.1, 126.5	66.5, 142.9
z score, mean (SD)	0.5 (1.0)	0.1 (0.9)	0.3 (1.0)
z score, range	-3.0, 3.5	-1.8, 2.3	-3.0, 3.5
FEV_1:			
% predicted, mean (SD)	102.9 (13.0)	95.4 (12.3)	99.6 (13.2)
% predicted, range	59.3, 130.1	74.5, 120.7	59.3, 130.2
z score, mean (SD)	0.3 (1.1)	-0.4 (1.1)	0.0 (1.1)
z score, range	-3.4, 2.3	-2.2, 1.8	-3.4, 2.3
FEF_{25-75}:			
% predicted, mean (SD)	90.9 (24.7)	80.3 (23.0)	86.2 (25.5)
% predicted, range	20.8, 184.8	38.9, 130.3	20.8, 184.8
z score, mean (SD)	-0.5 (1.2)	-1.0 (1.1)	-0.7 (1.2)
z score, range	-4.1, 3.6	-3.2, 1.2	-4.1, 3.6
FEV_1/FVC, mean (SD)	84.5 (6.9)	82.5 (6.6)	83.6 (6.8)
PEF_{spiro}, L/min, median (IQR)	267.3 (237.0, 332.7)	281.7 (221.7, 337.8)	266.7 (230.1, 336.9)

CHUSJ: Sainte-Justine hospital university center, CHUQ: Quebec hospital university center, FVC: forced vital capacity, $FEV_{1:}$ forced expiratory volume in 1 second, $FEF_{25-75:}$ forced expiratory flow 25-75%, FEV_1/FVC: forced expiratory volume in 1 second/forced vital capacity, PEF: peak expiratory flow, SD: standard deviation
